# Effect of Different Processing Methods on Phytochemical Contents and Neuroprotective Activity of *Camellia euphlebia* Leaves Extract

**DOI:** 10.1155/2019/1717090

**Published:** 2019-12-23

**Authors:** Dongye He, Shu Jia, Yongping Xu

**Affiliations:** ^1^Department of Central Laboratory, Affiliated Hospital of Jining Medical University, Jining 272000, China; ^2^School of Bioengineering, Dalian University of Technology, Dalian 116024, China

## Abstract

*Camellia euphlebia* is a new food source and traditional folk medicine in China. Previous studies have demonstrated the antidepressant activity of *Camellia euphlebia* extract by both *in vivo* and *in vitro* experiments. The effects of different pretreatments on phytochemical contents and neuroprotective activity of *Camellia euphlebia* extract were further investigated in order to develop an optimal processing method that makes the extraction more efficient. Six different powders of *Camellia euphlebia* leaves were prepared by different pretreatments. The particle size and morphology were examined by using a Malvern particle size analyzer and scanning electron microscopy, respectively. The results showed that the percentage of powder particle size within a range of 0.2∼40 *μ*m was up to 79.18% after press-shear assisted interaction technology pretreatment by 2% addition of shellfish shell powder, and the cells were broken completely. Additionally, the contents of flavonoids, polysaccharides, polyphenols, saponins, and catechin in the extract were 11.78 ± 0.62%, 34.60 ± 3.37%, 6.15 ± 0.29%, 9.43 ± 1.19%, and 1.99 ± 0.11%, respectively, which were higher than those of the other five extracts. Moreover, the extract had the strongest neuroprotective activity by comparing the neuroprotective effect of different extracts on corticosterone-induced neurotoxicity in differentiated PC12 cells. It is concluded that press-shear assisted interaction technology with 2% addition of shellfish shell powder pretreatment, to a great extent, improved the dissolution of bioactive ingredients in *Camellia euphlebia*.

## 1. Introduction

*Camellia euphlebia* Merr. Ex Sealy (Theaceae) is distributed naturally only in North Vietnam and Southwest China and documented by the Chinese medical classics Ben Cao Gang Mu (Compendium of Materia Medica) about 400 years ago and has been widely used to treat tumor, nephritis, hepatitis with jaundice, urinary tract infection, dysentery, hypertension, diarrhea, faucitis, and irregular menstruation. Importantly, the great potential in functional health products from *Camellia euphlebia* has attracted considerable attention since *Camellia euphlebia* was recognized as a new food source by the Ministry of Health in China in 2010 [1]. A wide variety of health-orientated tea and tea-based beverages have been produced with *Camellia euphlebia* flowers or leaves in China, and these include bag tea, instant tea, oral liquid, condensed liquid, tea essence, bud tea, and functional beverages, which are predominantly sold in Southeast Asia, Europe, and America. Our previous works also found that *Camellia euphlebia* aqueous extract (CEE) exhibited an antidepressant-like activity in several animal behavioral tests including tail suspension test, forced swimming test, and chronic unpredictable mild stress test via the alteration of hypothalamic-pituitary-adrenal axis (HPA) and monoaminergic systems *in vivo* [2, 3] and protected against corticosterone-induced apoptosis in differentiated PC12 cells by regulating the mitochondrial apoptotic pathway and PKA/CREB/BDNF signaling pathway *in vitro* [4]. Although these studies opened the possibility of *Camellia euphlebia* as an alternative dietary therapy to depression, the yield of CEE obtained from traditional processing methods is relatively low, thus directly hindering the development and utilization of this plant food resource. A major reason of low yield is that *Camellia euphlebia* leaf forms wax-rich cuticle and tubular network structure so that it is difficult to be pulverized completely. Therefore, natural bioactive ingredients imprisoned within plant cells cannot be fully released.

Press-shear assisted interaction (PAI) technology as a low-cost, high-efficient, and green processing technology has been widely used in the processing of medical plant materials or functional food products [5–7]. This technology can break down physical confinement of plant cell wall and organelles on bioactive ingredients, thereby improving the release of biologically active ingredients. Most importantly, this technology had been patented by Liu et al. in China [8]. In previous works, we found that the PAI combined with sharp brittle adjuvants could well pulverize plant materials with wax-rich cuticle and tubular network structure (data not shown). To screen a sample pretreatment method that can greatly improve the dissolution of active ingredients in *Camellia euphlebia*, we optimized the PAI by adding different proportions of shellfish shell powder as adjuvant and detected the particle size and morphology of different powders and the contents of the main active ingredients including flavonoids, polysaccharides, polyphenols, saponins, and catechins. Additionally, the neuroprotective activity of CEE prepared by different pretreatments was further evaluated.

## 2. Materials and Methods

### 2.1. Plant Material and Shellfish Shell Powder

Fresh leaves of *Camellia euphlebia* Merr. ex Sealy were obtained from Guangxi Zhuang Autonomous Region during its flowering period in 2017. The specimen was authenticated by Dr. Zhonghui Ma (Department of Botany Sciences, College of Agriculture, Guangxi University, China). A voucher specimen with number 8109255 has been deposited in the herbarium of Guangxi Institute of Botany, Chinese Academy of Sciences, China. Shellfish shell was obtained from Dalian Seafood Processing Factory, China. A certain amount of shellfish shells were washed for removing residual meat, attachments, etc. and were dried at room temperature. After being crushed into coarse pieces, shellfish shell was heated in a muffle furnace to 550°C for 1 h to remove the organic matter. Finally, the calcined coarse pieces were further crushed into 1–4 mm pieces by passing through 1 mm and 4 mm aperture sieves. The obtained shellfish shell powder has irregular concave-convex structure, and its main component is water-insoluble calcium carbonate (CaCO_3_). These physical and chemical properties of the shellfish shell powder ensure that it not only effectively collides with plant materials powder in the process of sample pretreatment but also does not interact with the phytochemical content of the leaves extract during ultrasonic-assisted water extraction.

### 2.2. Chemicals

Rutin, gallic acid, oleanolic acid, catechin, and glucose were purchased from Chengdu Must Biotechnology (Chengdu, China). DMEM and FBS were purchased from Gibco (Grand Island, NY, USA). Horse serum was obtained from Invitrogen (Carlsbad, CA, USA). Corticosterone (purity ≥ 98.5%) and NGF (purity ≥ 97%) were purchased from Sigma-Aldrich (St. Louis, MO, USA). Penicillin/streptomycin solution (100x) was supplied from Beyotime Biotechnology (Shanghai, China). MTT, DMSO, and 0.25% trypsin were purchased from Solarbio Life Sciences (Beijing, China). All other chemicals and reagents were of analytical grade.

### 2.3. Pretreatment Procedures

The fresh leaves were washed three times with tap water and then dried at 55°C for 6 h in a forced-air oven. The dried leaves were ground into a coarse powder with a pulverizer (HC-150T2, Yongkang Lv Ke Food Machinery Company, Zhejiang, China) at room temperature. 60 mesh powder and 200 mesh powder were prepared from the coarse powder by passaging through 0.25 mm and 0.075 mm aperture sieves, respectively. Additionally, the coarse powder was further processed by PAI as previously described [7, 9]. Briefly, as a processing adjuvant, shellfish shell powders with different mass ratios (0%, 1%, 2%, and 4%; w/w) were added to the coarse powder and four mixtures were ground into different ultrafine powders with an energy-intensive vibrational mill (WZJ-6J, Billion Power Engineering Co. Ltd., Jinan, China; mill volume = 1.2 L) equipped with a water-cooled drum and steel rods as grinding bodies to load weight (100 : 1), respectively. The mill was vibrated at a frequency of 16 Hz with steel rods acceleration of 9 g (g = 9.8 m/s^2^) and total power consumption of 0.75 kW. The prepared raw powders with different particle sizes were labeled as PS powder, PAI-1 powder, PAI-2 powder, and PAI-4 powder and stored in a sealed bag at −20°C until use, respectively.

### 2.4. Particle Size Analysis

The average particle sizes and size distributions of the six powders were measured by using a Malvern particle size analyzer (Nano ZS90, Malvern Instruments, UK). The particle size range for the analysis is 0.02∼1000 *μ*m. An appropriate amount of each sample was dispersed in suspension medium water as a dispersant agent, stirred at a pumping speed of 500 rpm. The more correct data were obtained by breaking up the flocculates ultrasonically. Then, the particle size and particle size distribution were further calculated from the light scattering pattern.

### 2.5. Scanning Electron Microscopy (SEM) Analysis

The morphology of the six powders prepared by different pretreatments was examined using SEM (Quanta 450, FEI, USA) at 600x, 1000x, and 2000x magnification. Samples were mounted on metal stubs, using double-sided adhesive tape, gold coated under vacuum condition, and then examined under high-vacuum condition with an accelerating voltage of 30 kV.

### 2.6. Determination of the Major Active Constituents

Preparation of CEE was performed as described in previous work. Six dried extracts were accurately weighed to calculate the extraction yields (%) and stored in a sealed bag at −20°C until use, respectively. The contents of the main active components in the six extracts prepared were quantitatively analyzed by UV-spectrophotometry. The contents of the total flavonoids, polysaccharides, polyphenols, saponins, and catechin were determined by the aluminum chloride [10], phenol-sulfuric acid [11], Folin-Ciocalteu [12], vanillin-glacial acetic acid-perchloric acid colorimetric [13], and vanillin-hydrochloric acid methods [14], respectively. Additionally, the contents of the total carbohydrate and reducing sugar were determined by the phenol-sulfuric acid and 3,5-dinitrosalicylic acid methods, respectively. The total polysaccharides content was calculated by the subtraction of reducing sugar from the total carbohydrate. Rutin, gallic acid, oleanolic acid, catechin, and glucose were used to generate the calibration curves.

### 2.7. Neuroprotective Activity

#### 2.7.1. Cell Culture

Rat pheochromocytoma PC12 cells were obtained from Cell Bank of Shanghai Institute of life Science (SCSP-517, Chinese Academy of Sciences, Shanghai, China) and were cultured in DMEM medium supplemented with 10% heat-inactivated FBS, 5% heat-inactivated HS, 100 U/mL penicillin, and 100 *μ*g/mL streptomycin at 37°C in a humidified atmosphere of 5% CO_2_ and 95% air. For differentiation, PC12 cells were grown on 25 cm^2^ plastic flask and kept in serum-containing medium for three to four days. Subsequently, to facilitate neurite outgrowth, cells were differentiated in 10% FBS medium supplemented with NGF (20 ng/mL) for seven to ten days. Fresh NGF was added every second day with the medium change. Differentiated PC12 cells were cultured in DMEM medium only supplemented with 10% heat-inactivated FBS and used for further experiments.

#### 2.7.2. Cell Viability Assay

The effect of extracts prepared by different pretreatments on differentiated PC12 cells viability was firstly investigated. Differentiated PC12 cells were seeded at a density of 5 × 10^3^ cells/well in a 96-well microplate for 32 h, and the cells were then treated with different concentrations (2.5, 5, 10, 20, 25, 50, 100, 200, 400, or 800 *μ*g/mL) of CEE for 24 h. Cell viability was determined by MTT assay. Furthermore, to test the protective effect of CEE against corticosterone-induced neurotoxicity, the differentiated PC12 cells were coincubated with 300 *μ*M of corticosterone and different concentrations of CEE (5, 10, 20, 40, 80, 160, and 320 *μ*g/mL) for 24 h. Likewise, cell viability was determined by MTT assay. Briefly, at the end of the treatment, the medium was carefully removed and fresh medium containing 0.5 mg/mL MTT was then added to the cells followed by 4 h incubation at 37°C. After that, the culture medium was replaced with an equal volume of DMSO followed by 10-minute incubation at room temperature. The absorbance of the plate was then measured at 570 nm by using a microplate reader (TECAN, Sunrise, Austria). Cell viability was expressed as percentage of control (nontreated) cells.

### 2.8. Statistical Analysis

Data were analyzed with the one-way analysis of variance followed by Tukey's *t*-test using GraphPad Prism 5.03 Software (GraphPad Software Inc., San Diego, CA, USA). The results were expressed as mean ± standard (SD). Differences between groups were considered to be statistically significant at the *P* < 0.05 level.

## 3. Results

### 3.1. Effect of Different Pretreatments on Particle Size

**Figure 1:**
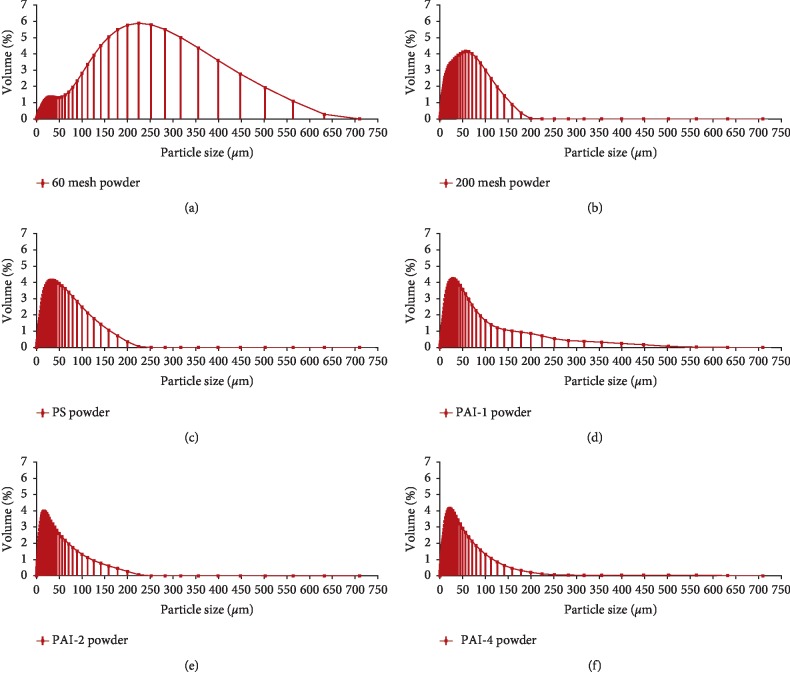
Particle size distribution of leaves powder from *Camellia euphlebia* powder processed by different pretreatments. PAI: press-shear assisted interaction technology.

Particle size distribution of leaves powder from *Camellia euphlebia* processed by different pretreatments was shown in Table 1. As shown in Figure 1, the maximum volume percentage of 60 mesh powder was almost 6%, and the corresponding particle size ranged up to 300 *μ*m. In contrast, the particle sizes corresponding to the maximum volume percentages of 200 mesh powder, PS powder, PAI-1 powder, PAI-2 powder, and PAI-4 powder were approximately 70 *μ*m, 40 *μ*m, 30 *μ*m, 25 *μ*m, and 18 *μ*m, respectively. Among these six powders, the maximum volume percentage of PAI-2 powder did not exceed 4% in the 0.2∼400 *μ*m size range. The results showed that the particle size of *Camellia euphlebia* powder prepared by PAI-2 became smaller and more uniform.

### 3.2. Effect of Different Pretreatments on Microstructural Changes

**Figure 2:**
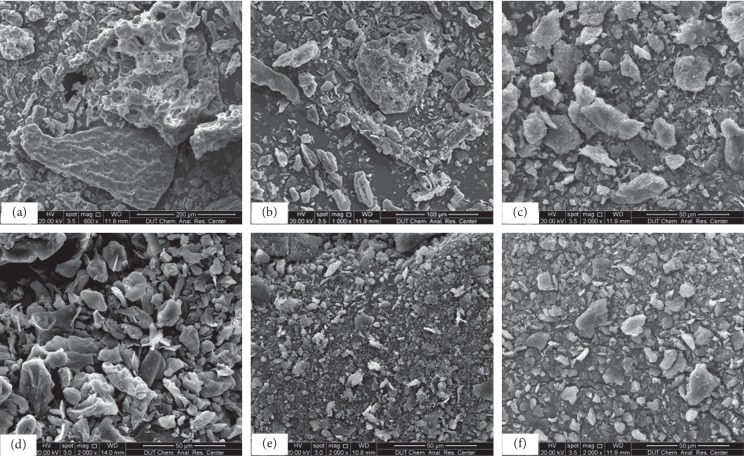
SEM micrographs of *Camellia euphlebia* processed by different procedures: (a) 60 mesh powder; (b) 200 mesh powder; (c) PS powder; (d) PAI-1 powder; (e) PAI-2 powder; (f) PAI-4 powder. PS: press shear technology; PAI: press-shear assisted interaction technology.

Compared with optical microscopy and transmission electron microscopy, SEM has wider field and higher resolution. SEM images (2000x) in Figure 2 showed intuitively that PAI-2 powder had a higher degree of comminution and more uniform particle size.

### 3.3. Effect of Different Pretreatments on Major Compositions

Obviously, PAI-2 pretreatments significantly increased the yield of aqueous extract, compared with other pretreatments (*P* < 0.05). Additionally, the contents of flavonoids, polyphenols, saponins, polysaccharides, and catechin in PAI-2 prepared CEE were higher than those of other pretreatments prepared CEE (Table 2). Among these major components, the contents of flavonoids from PAI-2 prepared CEE were 248.52%, 176.61%, 142.44%, 146.58%, and 230.53% of extracts from 60 mesh, 200 mesh, PS, PAI-1, and PAI-4, respectively. In addition, the contents of polyphenols, saponins, polysaccharides, and catechin from PAI-2 prepared CEE were at least 1.1 times more than those of 60 mesh, 200 mesh, PS, PAI-1, and PAI-4.

### 3.4. Effect of Extracts Prepared by Different Pretreatments on Differentiated PC12 Cells Viability

**Figure 3:**
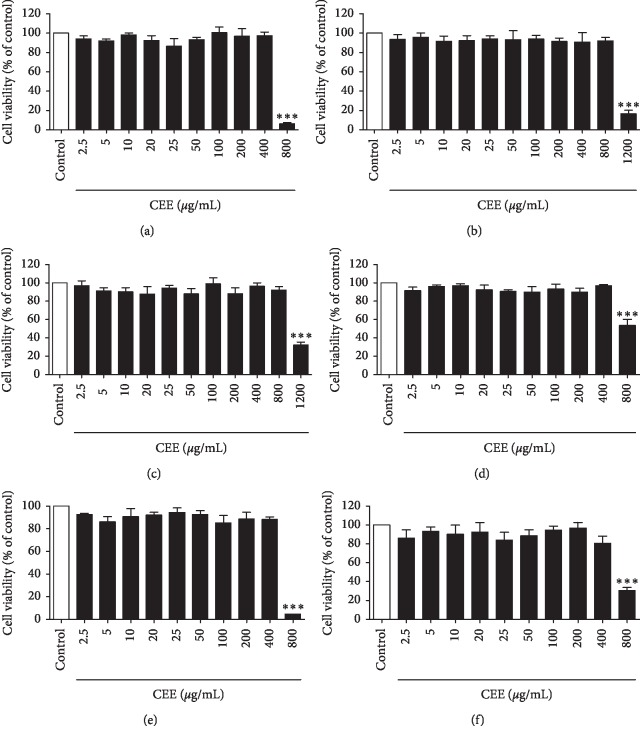
Effect of six extracts on cell viability in differentiated PC12 cells. Results are presented as means ± SEM (*n* = 5). ^*∗∗∗*^*P* < 0.001 as compared with control group. (a) 60 mesh extract; (b) 200 mesh extract; (c) PS powder extract; (d) PAI (1%) powder extract; (e) PAI (2%) powder extract; (f) PAI (4%) powder extract.

As shown in Figure 3, compared with control group, 2.5∼400 *μ*g/mL CEE prepared from 60 mesh powder, 200 mesh powder, PS powder, PAI-1 powder, PAI-2 powder, and PAI-4 powder exhibited no significant effect on cell viability in differentiated PC12 cells (*P* > 0.05), suggesting that the safe dose range of these extracts was less than 400 *μ*g/mL. Therefore, the dose gradient was set at 5, 10, 20, 40, 80, 160, and 320 *μ*g/mL, and these doses would be used for subsequent experiments.

### 3.5. Effect of Different Pretreatments on Neuroprotective Activity of CEE

**Figure 4:**
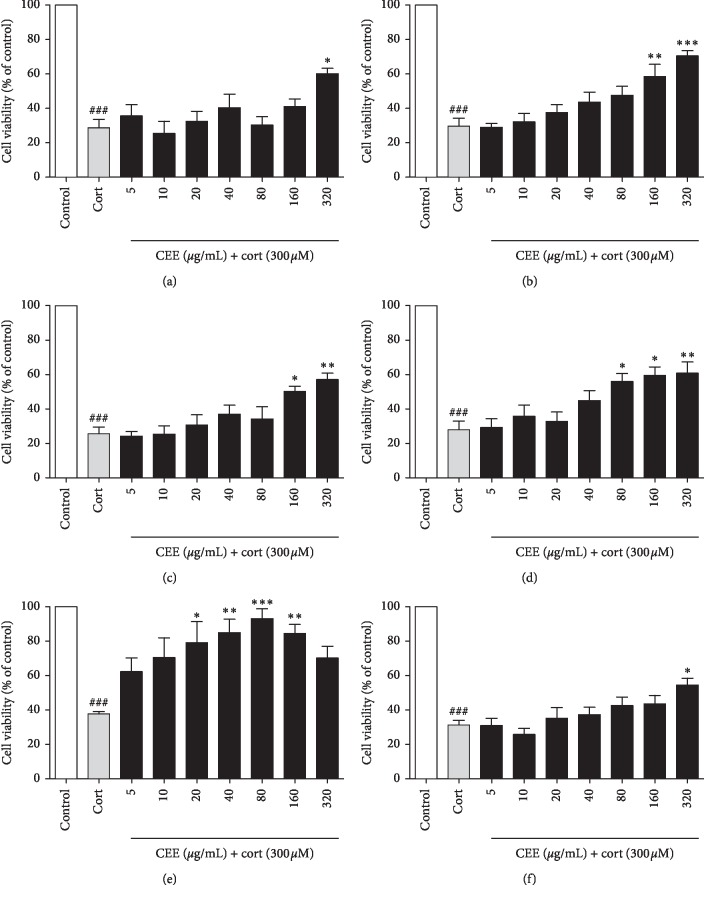
Effect of six extracts on cell viability in corticosterone-treated differentiated PC12 cells by MTT assay. ^###^*P* < 0.001 as compared with control group; ^*∗*^*P* < 0.05, ^*∗∗*^*P* < 0.01, or ^*∗∗∗*^*P* < 0.001 as compared with corticosterone-treated group. (a) 60 mesh extract; (b) 200 mesh extract; (c) PS powder extract; (d) PAI-1 powder extract; (e) PAI-2 powder extract; (f) PAI-4 powder extract.

The results obtained from MTT assay showed that, compared with control group, treatment with 300 *μ*M of corticosterone for 24 h had been shown to cause cytotoxicity in PC12 cells, as evidenced by approximately 70% cell viability loss. However, 320 *μ*g/mL of CEE prepared by 60 mesh powder and PAI-4 powder, in the presence of 300 *μ*M corticosterone, significantly increased the cell viability by 60.10 ± 5.33% (*P* < 0.05) and 54.43 ± 6.98% (*P* < 0.05), respectively (Figures 4(a) and 4(f)). Additionally, different concentrations of CEE (5, 10, 20, 40, 80, 160, and 320 *μ*g/mL) prepared by 200 mesh powder, PS powder, and PAI-1 powder, respectively, could inhibit the corticosterone-induced neurotoxicity with a dose-dependent manner in differentiated PC12 cells (Figures 4(b)–4(d)). In contrast, PAI-2 powder prepared CEE had the strongest neuroprotective effect and its minimum effective concentration was 20 *μ*g/mL. Compared with corticosterone-treated group, different concentrations of CEE prepared by PAI-2 powder (20, 40, and 80 *μ*g/mL) significantly increased the cell viability by 79.08 ± 21.17% (*P* < 0.05), 84.90 ± 15.54% (*P* < 0.01), and 93.08 ± 11.42% (*P* < 0.05), respectively (Figure 4(e)). Interestingly, 160 *μ*g/mL of this extract only increased the cell viability by 84.43 ± 9.11% (*P* < 0.01), suggesting that its neuroprotective effect began to weaken when its dose was more than 80 *μ*g/mL.

## 4. Discussion

PAI is a promising biotechnology that produces high-intensity and high-frequency interaction between plant material, adjuvant, and machine by means of physical and mechanical actions. This technology has been widely used in the extraction of plant active substances or functional foods, and many relevant studies have been carried out by our team in this field. For example, Li processed *Capsicum annuum* using PAI combined with HP-*β*-CD as an adjuvant and found that the extraction rate and average color value of capsaicin were significantly increased by 28.16% and 18.11%, respectively, when compared with the traditional heat-reflux extraction method [15]. Another study has demonstrated that PAI combined with Na_2_CO_3_ or Na_2_B_4_O_7_ as an adjuvant effectively increased the extraction yield of flavonoids in *Acanthopanax senticosus* by 13.2%, as compared with the traditional heat-reflux extraction method [4]. It was worth mentioning that the selection of physical or chemical adjuvants depends on the physicochemical properties of the extracted substances and structural characteristics of plant materials. In the present study, considering the wax-rich cuticle and tubular network structure of *Camellia euphlebia* leaves, we applied shellfish shell powder as a physical adjuvant in the process of sample pretreatment. The results showed that the percentage of particle size of *Camellia euphlebia* leaves powder within a range of 0.2∼40 *μ*m was up to 79.18% after PAI pretreatment with the addition of 2% shellfish shell powder, which significantly increased by 3.72 times and 1.28 times more than the 60 mesh powder and 200 mesh powder prepared by traditional methods, respectively (Table 1). Additionally, the elevated yield of the extraction and content of major constituents including flavonoids, polyphenols, saponins, polysaccharides, and catechin further proved the high efficiency of this method in the extraction of CEE (Table 2).

Corticosterone-induced neurotoxicity in differentiated PC12 cells has extensively used the *in vitro* model for screening the natural products with neuroprotective activity [16–18]. Therefore, the present study applied this *in vitro* cell model to evaluate the neuroprotective activities of *Camellia euphlebia* extracts prepared from 60 mesh, 200 mesh, PS powder, PAI-1 powder, PAI-2 powder, and PAI-4 powder (Figure 4), in order to evaluate the effect of different processing methods on the neuroprotective activity of *Camellia euphlebia* extract. The results demonstrated that PAI-2 powder prepared CEE had the strongest neuroprotective activity and its minimum effective concentration was 20 *μ*g/mL. Taken together, CEE prepared by PAI-2 had the highest contents of several major ingredients, as well as the strongest neuroprotective activity, indicating that high dissolution of internal active ingredients of CEE caused by PAI-2 inevitably increased the number and content of active ingredients with neuroprotective activity.

## 5. Conclusions

In summary, our research showed that the PAI method optimized by adding 2% shellfish shell powder made the extraction of CEE more efficient. CEE prepared by PAI-2 pretreatment had notable advantages of higher yield, phytochemicals with higher dissolution, and stronger neuroprotective activity and represented a more valuable alternative to the traditional method for the preparation of functional plant food products.

## Figures and Tables

**Table 1:** Particle size distribution of leaves powder from *Camellia euphlebia* processed by different pretreatments.

Sample^*∗*^	Sample size distribution (%)		
	0.2–40 *μ*m	40–200 *μ*m	200–1000 *μ*m
60 mesh	21.31	42.39	36.30
200 mesh	62.00	38.00	0.00
PS	65.20	34.74	0.06
PAI-1	68.42	28.70	2.88
PAI-2	79.18	20.74	0.08
PAI-4	77.81	21.69	0.50

^*∗*^PS: press shear technology; PAI: press-shear assisted interaction technology.

**Table 2:** : Comparison of the major components of aqueous extracts processed by different pretreatments.

Samples	Yield of extracts (%)	Phytochemical contents (%)^*∗*^				
		Flavonoids	Polyphenols	Saponins	Polysaccharides	Catechin
60 mesh	10.40 ± 0.79^a^	4.74 ± 0.68^a^	2.64 ± 0.71^a^	5.09 ± 0.80^a^	18.50 ± 1.50^a^	0.53 ± 0.04^a^
200 mesh	11.93 ± 0.40^b^	6.67 ± 0.18^ab^	5.59 ± 0.93^b^	7.40 ± 0.78^ab^	25.40 ± 1.98^ab^	1.33 ± 0.07^b^
PS	12.83 ± 0.57^b^	8.27 ± 1.18^bc^	3.72 ± 0.73^a^	7.22 ± 0.0.90^ab^	24.85 ± 3.34^ab^	1.54 ± 0.09^bc^
PAI-1	16.90 ± 0.44^c^	8.05 ± 0.82^bc^	5.45 ± 0.52^b^	7.65 ± 0.93^ab^	30.93 ± 5.09^bc^	1.40 ± 0.08^bc^
PAI-2	18.93 ± 0.25^d^	11.78 ± 0.62^d^	6.15 ± 0.29^b^	9.43 ± 1.19^b^	34.60 ± 3.37^c^	1.99 ± 0.11^d^
PAI-3	15.73 ± 0.60^c^	5.11 ± 0.46^a^	5.75 ± 0.60^b^	6.26 ± 1.01^a^	27.10 ± 3.08^abc^	1.57 ± 0.0.05^c^

^*∗*^The values are expressed as mean ± SEM from triplicate assays. Within each row, values with different superscripts are significantly different from each other (*P* < 0.05), as determined by Tukey's *t*-test. PS: press shear technology; PAI: press-shear assisted interaction technology.

## Data Availability

The data used to support the findings of this study are available from the corresponding author upon request.
